# Knowledge, attitudes and practices relating to antibiotic use and resistance among prescribers from public primary healthcare facilities in Harare, Zimbabwe

**DOI:** 10.12688/wellcomeopenres.16657.2

**Published:** 2022-04-29

**Authors:** Ioana D. Olaru, Rashida A. Ferrand, Shunmay Yeung, Rudo Chingono, Prosper Chonzi, Kudzai P.E. Masunda, Justin Dixon, Katharina Kranzer

**Affiliations:** 1 Department of Clinical Research, London School of Hygiene & Tropical Medicine, London, WC1E 7HT, UK; 2 Biomedical Research and Training Institute, Harare, Zimbabwe; 3 Department of Paediatric Infectious Diseases, St Mary’s Imperial College Hospital, London, W2 1NY, UK; 4 Department of Health, Harare City Council, Harare, Zimbabwe; 5 Department of Global Health and Development, London School of Hygiene & Tropical Medicine, London, WC1E 7HT, UK; 6 Division of Infectious and Tropical Diseases, Medical Centre of the University of Munich, Munich, 80802, Germany

**Keywords:** AMR, antibiotic resistance, antibiotic use, outpatients

## Abstract

Background

Overuse of antibiotics is one of the main drivers for antimicrobial resistance (AMR). Globally, most antibiotics are prescribed in the outpatient setting. This survey aimed to explore attitudes and practices with regards to microbiology tests, AMR and antibiotic prescribing among healthcare providers at public primary health clinics in Harare, Zimbabwe.

Methods

This cross-sectional survey was conducted in nine primary health clinics located in low-income suburbs of Harare between October and December 2020. In Zimbabwe, primary health clinics provide nurse-led outpatient care for acute and chronic illnesses. Healthcare providers who independently prescribe antibiotics and order diagnostic tests were invited to participate. The survey used self-administered questionnaires. A five-point Likert scale was used to determine attitudes and beliefs.

Results

A total of 91 healthcare providers agreed to participate in the survey. The majority of participants (62/91, 68%) had more than 10 years of work experience. Most participants reported that they consider AMR as a global (75/91, 82%) and/or national (81/91, 89%) problem, while 52/91 (57%) considered AMR to be a problem in their healthcare facilities. A fifth of participants (20/91, 22%) were unsure if AMR was a problem in their clinics. Participants felt that availability of national guidelines (89/89, 100%), training sessions on antibiotic prescribing (89/89, 100%) and regular audit and feedback on prescribing (82/88, 93%) were helpful interventions to improve prescribing.

Conclusions

These findings support the need for increased availability of data on AMR and antibiotic use in primary care. Educational interventions, regular audit and feedback, and access to practice guidelines may be useful to limit overuse of antibiotics.

## Introduction

Global antibiotic consumption has increased by more than 65% within the last two decades, driven primarily by an increase in consumption in low- and middle-income countries (LMICs) and reflecting economic growth ^1^. Inappropriate antibiotic use is frequent in many settings with at least 30% of all antibiotic prescriptions considered inappropriate ^2– 4^. This has public health implications since antibiotic overuse is one of the major drivers for antimicrobial resistance (AMR) ^5^.

In high-income countries, more than 85% of antibiotics are prescribed in the community i.e. in outpatient settings ^6^; this is likely similar in LMICs. One in eight and one in two outpatient consultations result in antibiotic prescriptions in high and low-income settings, respectively ^3, 7^. This difference may be explained by the higher prevalence of infectious diseases and a lack of access to diagnostic testing. In addition, the high workload in low-resource outpatient settings may lead to reduced consultation time and increase the likelihood of antibiotic prescriptions ^8, 9^. In many low-resource settings, non-prescription antibiotic use is a frequent phenomenon ^10^. In Zimbabwe, antibiotic dispensing was historically highly regulated with only 8% of antibiotics issued without a prescription ^11^. However, recent economic decline, increasing healthcare utilisation costs and the COVID-19 pandemic, have likely resulted in increased non-prescription antibiotic use ^12^.

While there are available data particularly on the prescribing practices of doctors working in hospitals, data from outpatient settings in LMICs where nurses are the main antibiotic prescribers are scarce. A better understanding of attitudes and practices of healthcare providers relating to AMR and antibiotic use may allow for the development of strategies to improve prescribing and ultimately curb the increase in AMR. This survey aimed to explore attitudes and practices with regards to microbiology tests, AMR and antibiotic prescribing among healthcare providers (nurses and midwives) at public primary health clinics in Harare, Zimbabwe.

## Methods

### Setting

Primary health clinics (PHCs) provide nurse-led care for acute and chronic illnesses including HIV and non-communicable diseases as well as antenatal and maternity services for uncomplicated deliveries and well-child clinics for growth monitoring and immunisations. Microbiology diagnostic services beyond rapid testing for malaria and HIV are only available at central laboratories. Pharmacies co-located on PHC premises fill prescriptions at reduced costs compared to independent pharmacies however, stock-outs of medicines are frequent. Unlike in many other countries, in Zimbabwe, most patients have to pay out-of-pocket for healthcare costs such as consultations, diagnostic tests and prescriptions, limiting access to care. In addition, Zimbabwe has been facing considerable hardships in recent years due to economic decline and rapid inflation which impacted on healthcare access and provision.

### Study design and participants

This cross-sectional survey was conducted in nine PHCs located in low-income suburbs of Harare between October and December 2020. The PHCs were selected out of 12 facilities if they were serving a low-income population in southern Harare and if they were operational at the time of the survey. Healthcare providers who independently prescribe antibiotics and order diagnostic tests (e.g. nurses, midwives, etc.) were eligible to participate in the survey. The surveys were conducted before dissemination and feedback sessions discussing the results of two studies focusing on viral and bacterial infections and AMR ^13, 14^. All healthcare workers who were working at the clinic on the day of the survey were invited to participate. The clinic matrons were informed about the dissemination sessions and the plan to conduct the survey and provided their support.

### Survey

The survey ^15^ was developed based on a literature review ^8, 16– 18^ and findings from other studies conducted in Zimbabwe ^13, 14, 19^. The studies did not assess the knowledge, attitudes and practices of healthcare workers but rather provided a more comprehensive understanding of the landscape of AMR and prescribing in Zimbabwe. Data on demographics, training and work experience were collected. Main topics addressed by the questions were: availability and use of diagnostic tests that may be used to identify infections with antibiotic resistant pathogens; pathogens encountered in current practice; attitudes and beliefs relating to AMR and antibiotic prescribing; training and sources of information used to improve prescribing. Most questions used a five-point scale with the exception of demographics and questions on the importance of AMR and on sources of information. Questions were answered in terms of importance (very important to very unimportant), helpfulness (very helpful to very unhelpful), and agreement of the survey taker with a particular statement (strongly agree to strongly disagree) (see extended data for survey and codebook ^15^). Knowledge about diagnostic testing and drug susceptibility testing was evaluated using four multiple-choice and free-text questions. The clinical questions were selected to reflect common scenarios that the nurses would encounter in their daily practice and might lead to inappropriate antibiotic use.

### Data collection

Data was collected as part of the ARGUS study which evaluates gram-negative resistance and antibiotic usage in primary care ^13^. Ethical approval was obtained from the Medical Research Council Zimbabwe (MRCZ/A/2406) and the London School of Hygiene and Tropical Medicine Ethics committee (Ref. 16424).

All prescribers who were working at the clinics on the day of the event were invited to take part in the survey. Each clinic was visited once. The survey contained an information sheet on the purpose of the survey and consent. This section specifically asked the participants to fill in and return the survey if they consented to participate. Data was fully anonymised on collection and no participant identifiers were used. The questionnaires were self-administered using paper-based forms and were filled in prior to the session. Data from the paper questionnaires was entered into electronic forms using Open Data Kit (ODK).

### Statistical analysis

Data analysis was performed in R v4.0.3 (The R Project for Statistical Computing). Categorical variables were presented as counts and percentages. A five-point Likert scale was used to determine attitudes and beliefs ranging from 1 point (“very important”, “very helpful”, “strongly agree”) to 5 points (“very unimportant”, “very unhelpful”, “strongly disagree”). Results were presented aggregated for positive and negative categories (e.g. very important and important formed one category). For these questions, percentages were calculated while excluding questions which were unanswered or where the response was “do not know”. To account for non-response, the denominator for the data is reported.

## Results

**Figure 1.**
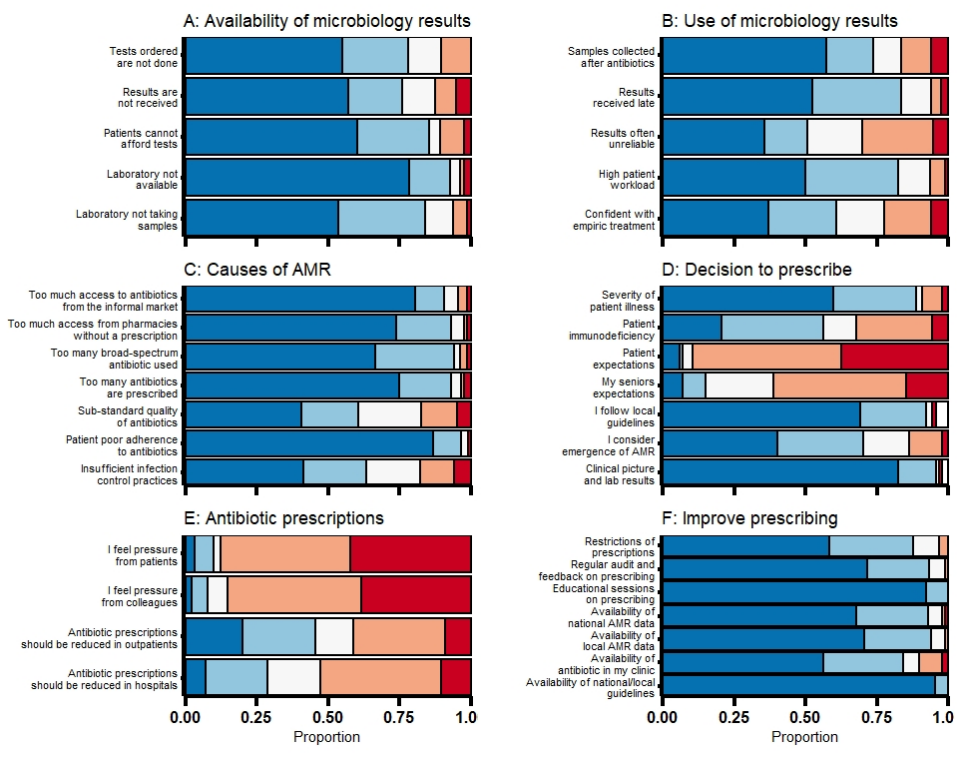
Attitudes and practices relating to microbiology tests, antimicrobial resistance and antibiotic prescriptions. Positive responses are displayed in blues, negative in reds and neutral responses in white. ( **A**) affecting the availability of microbiology testing (very important to very unimportant); ( **B**) affecting the use of microbiology results (very important to very unimportant); ( **C**) causes of AMR (very important to very unimportant); ( **D**) guiding the decision to start antibiotics (strongly agree to strongly disagree); ( **E**) antibiotic prescriptions (strongly agree to strongly disagree); ( **F**) improving antibiotic prescribing (very helpful to very unhelpful).

A total of 91 healthcare providers from nine PHCs were approached and all agreed to participate in the survey ^15^. Most participants (81/91, 89%) were female and worked in public health facilities only (84/91, 92%), while seven also worked in private health facilities or hospitals. Participants were senior nurses (44/91, 49%), midwives (34/91, 37%), and junior nurses (12/91, 13%). The majority of participants, 62/91 (68%) had more than 10 years of work experience. Figure 1 shows the attitudes and beliefs of healthcare providers related to diagnostic testing, causes of AMR and antibiotic prescribing.

### Microbiology test availability and use

Among 69 participants who reported having ordered specific microbiology tests within the previous month, 67/69 (97%) reported ordering a sputum test for tuberculosis with 19/67 (28%) having ordered more than 10 tests for tuberculosis. Urine cultures were ordered by 46/69 (67%) and stool cultures by 31/69 (45%) with 13/46 (28%) and 7/31 (23%) ordering more than five tests in the previous month, respectively. The main challenges in ordering and performing microbiology tests were the lack of access to laboratory testing (78/84, 93%), delays in receiving test results (70/84, 83%), high patient volume (66/80, 83%) and costs of testing (71/83, 86%; Figure 1A and 1B).

### Antimicrobial resistance

Most participants reported that they consider AMR as a global (75/91, 82%) and/or national (81/91, 89%) problem, while 52/91 (57%) considered AMR to be a problem in their healthcare facilities. A fifth of participants (20/91, 22%) were unsure if AMR was a problem in their clinics. Among key pathogens, 73/91 (80%), 45/91 (49%), 9/91 (10%) and 8/91 (9%) considered drug resistance to be a problem in *Mycobacterium tuberculosis*, *Salmonella* Typhi, *Staphylococcus aureus* (methicillin-resistant) and gram negatives (presence of extended-spectrum beta-lactamases), respectively. Poor adherence of patients to prescribed antibiotics treatment (87/90, 97%), over-prescription of antibiotics (82/88, 93%) and excessive use of unregulated antibiotics acquired from pharmacies without a prescription (82/88, 93%) or from the informal market (79/87, 91%) were considered very important or important drivers of AMR ( Figure 1C).

### Antibiotic prescribing

The decision to prescribe antibiotics was mainly influenced by the clinical presentation and laboratory results (87/89, 98%) and severity of illness (79/89, 89%) and was guided by the national guidelines ^20^ (84/87, 97%; Figure 1D). The decision to prescribe antibiotics was influenced by the patients’ or their seniors’ expectations in 6/88 (7%) and 13/88 (15%), respectively. Respondents reported prescribing unnecessary antibiotics very often (7/90, 8%), often (8/90, 9%), about half of the times to (29/90, 32%), sometimes (27/90, 30%) and almost never (19/90, 21%). In total, 25 (29%) and 41/90 (46%) of prescribers felt that antibiotic prescriptions should be reduced for inpatients and outpatients, respectively ( Figure 1E).

National guidelines were the main source for guiding prescribing in routine practice (85/91, 93%) and as a means to increase knowledge on antibiotic prescribing (88/91, 97%). Other sources of information to support prescribing were textbooks in 64/91 (70%), discussions with colleagues 57/91 (63%) and professional meetings 56/91 (62%). A third of participants (28/91, 31%) reported having received training in antibiotic prescribing in the previous year. Participants felt that availability of national guidelines (89/89, 100%), training sessions on antibiotic prescribing (89/89, 100%) and regular audit and feedback on prescribing (82/88, 93%) were helpful interventions to improve prescribing ( Figure 1F).

### Prescriber knowledge

Among survey participants, 84/91 (92%) would order a sputum test for tuberculosis in a patient with a prolonged cough and 71/91 (78%) would prescribe appropriate antibiotics in a patient with typhoid fever symptoms. In total, 18 (20%) would prescribe inappropriate antibiotics such as kanamycin and doxycycline to a pregnant patient with symptoms of a sexually transmitted infection. Most participants (81/91, 89%) would prescribe antibiotics in a patient with symptoms suggestive of a viral respiratory tract infection.

## Discussion

The study used a new approach by focusing on nurses and midwives from PHCs who are the main prescribers in the outpatient setting in Zimbabwe. This study found that although healthcare providers were aware of the challenges posed by AMR on a global and national level, they considered it less of an issue in their daily practice. Furthermore, while over-prescription of antibiotics was recognized as a problem by most, half of the participants reported that unnecessary prescriptions are infrequent in their current practice. These issues may arise from insufficient knowledge of the prevalence of AMR in their specific setting and from the propensity to attribute it to factors outside their own practice which is also reported by studies elsewhere ^21^. This may also come from the perception of futility that their daily practice will impact on AMR on a national or global level ^22^. Only one in three participants reported having received formal training on antibiotic prescribing in the previous year.

Limited availability of diagnostics, insufficient laboratory capacity and high costs of diagnostics means that most patients accessing outpatient departments in sub-Saharan Africa are treated using a “syndromic approach” ^23^. This was also reflected by the findings of this survey where healthcare providers reported that there are a number of barriers in accessing microbiological testing such as the lack of access to laboratory testing and high costs which are incurred by the patients. The use of microbiology tests plays an important role in bacterial identification and antibiotic susceptibility testing. Limiting tests to complex cases and patients presenting to private healthcare facilities will lead to data which may not reflect the burden of AMR in the community. Therefore, insufficient laboratory testing results in inadequate and potentially biased surveillance data thus preventing the development of setting-specific treatment recommendations.

Most survey participants were aware of resistance in *M. tuberculosis* likely due to the roll-out and decentralisation of testing using GeneXpert and awareness campaigns on the importance of tuberculosis diagnosis. Resistance in *S.* Typhi was often reported, reflecting the extensive information on the ongoing typhoid fever outbreak ^24^ provided to healthcare workers by overseeing authorities and non-governmental organizations. Conversely, less than 10% of respondents cited resistance in key pathogens such as methicillin resistance in *S. aureus* and the production of extended-spectrum beta-lactamases in *Enterobacteriaceae*. This may be related to the setting of the survey in outpatient facilities and to limited antibiotic susceptibility testing making the identification of these pathogens infrequent in daily practice. Furthermore, there may be a lack of published and widely disseminated information leading to decreased awareness among healthcare workers.

Most healthcare providers indicated that the decision to prescribe antibiotics is mainly guided by the clinical presentation and the national guidelines and not directly by patient expectations. This is reassuring and contrary to findings from other settings where patients’ expectations played an important role in the decision to prescribe antibiotics ^3, 16, 18^. However, there may be indirect pressures on the healthcare worker because they are aware that the patient may not be able to afford accessing the clinic again if symptoms become worse ^7^. Furthermore in this study, the national guidelines were described as the main “influencer” in guiding antibiotic prescribing in routine practice. This is in contrast to a study from Gabon showing that prior experience and the opinion of the superior strongly influenced the decision whether or not antibiotics should be prescribed ^25^.

A total of nine out of ten healthcare workers felt that antibiotics are overused in the formal sector contributing to the increase in AMR. This is a common finding globally ^8, 17^. Challenges in accessing healthcare such as clinic consultation fees for subsequent visits and potential hospital costs in case of clinical deterioration, promote the prescription of potentially unnecessary antibiotics “just in case” ^7^. Generally, healthcare workers will likely prioritise the potential immediate impact of antibiotic prescribing on individual patient outcome over the long-term effects of overuse on AMR on a population-level ^22^. Furthermore, in this survey, healthcare workers indicated that antibiotics purchased over the counter from pharmacies or informal vendors may facilitate development of AMR in their communities, highlighting the major challenge of unregulated drug use in LMICs ^10, 26^. Prescription-drugs in Zimbabwe have historically been very well regulated in comparison to its neighbours, with few non-prescription sales documented in multi-country surveys ^10^. However, starting from the economic crisis in 2007, the informal sector grew considerably, including an increase in informal vendors for antibiotics ^12^.

Regarding strategies to improve antibiotic prescribing, healthcare workers favoured educational and decision support measures such as training and increased availability of guidelines and prescribing data for their setting over restrictive measures for improving prescribing in their daily practice. These may represent effective strategies to improve prescribing as shown in other settings ^27, 28^.

To our knowledge, this is the first survey evaluating the attitudes and practices relating to AMR and antibiotic use among healthcare providers working in PHCs in Zimbabwe. Furthermore, the approach to survey nurses and midwives who are the main antibiotic prescribers in the public sector for outpatients in many settings is innovative. The findings of this survey are of particular importance and can be used to inform the design of future educational activities for this group of healthcare professionals working in PHCs in Zimbabwe and elsewhere. This study has several limitations. As the data were collected within a survey, participants may have given socially-desirable answers. In the attempt to avoid this, data collection was completely annonymous. Only 30% of respondents reported having received training in the previous year and 7% reported that their decision to prescribe antibiotics was based on their seniors’ expectations suggesting that responses were not given according to social desirability and supporting the validity of our findings. The study included a relatively small number of participants. However, all prescribers working on the day of the survey across nine PHCs in Harare were invited to participate with no refusals recorded, making the data generalizable to public sector providers of outpatient care in Harare. However, these findings may not be generalizable to healthcare workers working in private clinics or rural settings. Participants may have misunderstood some of the questions however the questions were informed by questionnaires used in other studies from sub-Saharan Africa and responses were generally consistent. Also, responses to some questions may be difficult to interpret because the respondent may have answered in the same way if they agreed with a statement of thought it was important. While increased availability of diagnostics is desirable, roll out is challenged by financial and infrastructural constraints. Also, in reality, turnaround times of microbiological diagnostics is usually too long and hence has limited impact on patient management, specifically in outpatient settings. However, establishing sentinel sites to determine causative organisms in certain settings and generate data on AMR might be a possible solution. In many settings nurses and midwives are the main antibiotic prescribers. Hence understanding how to design training programmes aimed at nurses and midwives and how to communicate AMR surveillance data to them is important. Surveys such as the one presented in this study conducted in other settings could potentially guide training and teaching programmes.

## Data availability

### Underlying data

DRYAD: Knowledge, attitudes and practices relating to antibiotic use and resistance among prescribers from public primary healthcare facilities in Harare, Zimbabwe. https://doi.org/10.5061/dryad.66t1g1k1s.

This project contains the following underlying data:

- Raw answers to survey

### Extended data

DRYAD: Knowledge, attitudes and practices relating to antibiotic use and resistance among prescribers from public primary healthcare facilities in Harare, Zimbabwe. https://doi.org/10.5061/dryad.66t1g1k1s.

This project contains the following extended data:

- Data codebook
- Survey questionnaire
- STROBE checklist

Data are available under the terms of the Creative Commons Zero "No rights reserved" data waiver (CC0 1.0 Public domain dedication).
