# Supplementary material for: Knowledge, attitudes and practices relating to antibiotic use and resistance among prescribers from public primary healthcare facilities in Harare, Zimbabwe
Source: Wellcome Open Res. 2022 Apr 29;6:72. Originally published 2021 Mar 30. [Version 2] doi: 10.12688/wellcomeopenres.16657.2 (PMC10534082; doi:10.12688/wellcomeopenres.16657.2)
Supplement: Peer review history

# Reviewer report: Reviewer response for version 2

**DOI:** 10.21956/wellcomeopenres.19797.r65873

**Published:** 2023-09-27

**Authors:** Nkuchia M. M'ikanatha

**Affiliations:** 1 Pennsylvania Department of Health, Harrisburg, PA, USA

This manuscript reports the results of a cross-sectional survey regarding knowledge and attitudes about antimicrobial resistance (AMR) among healthcare providers in the suburbs of Harare, Zimbabwe. Close to sixty percent of the healthcare providers considered AMR a threat in their facilities while most (over 80 percent) expressed a need for guidelines and training. Healthcare providers were also interested in regular audits and feedback on prescribing.

 The manuscripts provide main steps including background, methods, results, discussion, and conclusions.

 The generalizability of the study can be strengthened by comparing these results with what is known in Zimbabwe and the region. The author can provide some pertinent background—The population of Harare in 2020 was about 1.5 million. What was the estimated population served by the nine primary care clinics?  Is training on antimicrobial prescribing done annually, are there restrictions on the nurses and midwives who can prescribe? Putting these data in the African context by comparing what the authors found and what is known in countries in Southern Africa, for example, Botswana, and even Kenya in East Africa would be helpful.

 The authors can also provide some details on how the questions were developed—including the survey (as an appendix) would help the reader. Comments on the study limitations are necessary–such as the potential clinic selection bias and the days when interviews were conducted. It is surprising that the manuscript is silent about the COVID-19 pandemic. The impact of COVID-19 should be briefly described, and if there was none, let the reader know.  It would also be helpful to comment on how these findings relate to Zimbabwe’s national action plan for AMR.

 The structure of the manuscript can be improved by moving some of the details in the methods to the background section, for example, the statement, “Zimbabwe has been facing considerable hardships in recent years due to economic decline and rapid inflation which impacted on healthcare access and provision.”

Is the work clearly and accurately presented and does it cite the current literature?

Yes

If applicable, is the statistical analysis and its interpretation appropriate?

Not applicable

Are all the source data underlying the results available to ensure full reproducibility?

Partly

Is the study design appropriate and is the work technically sound?

Yes

Are the conclusions drawn adequately supported by the results?

Partly

Are sufficient details of methods and analysis provided to allow replication by others?

Partly

Reviewer Expertise:

Surveillance for antimicrbial resistance

I confirm that I have read this submission and believe that I have an appropriate level of expertise to confirm that it is of an acceptable scientific standard, however I have significant reservations, as outlined above.

# Reviewer report: Reviewer response for version 2

**DOI:** 10.21956/wellcomeopenres.19797.r50256

**Published:** 2022-05-12

**Authors:** Heiman F. L. Wertheim, Annelie Monnier

**Affiliations:** 1 Division of Medical Microbiology and Radboudumc Center for Infectious Diseases, Radboud University Medical Center, Nijmegen, The Netherlands; 2 Radboud University Medical Center, Nijmegen, The Netherlands

The comments have been adequately adressed.

Is the work clearly and accurately presented and does it cite the current literature?

Partly

If applicable, is the statistical analysis and its interpretation appropriate?

Yes

Are all the source data underlying the results available to ensure full reproducibility?

Yes

Is the study design appropriate and is the work technically sound?

Partly

Are the conclusions drawn adequately supported by the results?

Partly

Are sufficient details of methods and analysis provided to allow replication by others?

Partly

Reviewer Expertise:

NA

We confirm that we have read this submission and believe that we have an appropriate level of expertise to confirm that it is of an acceptable scientific standard.

# Reviewer report: Reviewer response for version 1

**DOI:** 10.21956/wellcomeopenres.18368.r49039

**Published:** 2022-03-30

**Authors:** Heiman F. L. Wertheim, Annelie Monnier

**Affiliations:** 1 Division of Medical Microbiology and Radboudumc Center for Infectious Diseases, Radboud University Medical Center, Nijmegen, The Netherlands; 2 Radboud University Medical Center, Nijmegen, The Netherlands

**Summary:**

 The authors report on a cross-sectional survey exploring attitudes and practices of health care providers working in public primary health clinics in relation to microbiology tests, AMR and antibiotic use, in the Harare region in Zimbabwe. The choice of the research setting is especially of interest as nurses are the main antibiotics prescribers in public health clinics and literature on nurse prescribers is particularly scarce. Minor revisions would benefit the clarity of the manuscript and highlight the novelty of the work and thereby enhance its impact.

**Major Comments:**

- Not applicable.

**Minor Comments:**

- Abstract: Last sentence of the methods section: 'to determined’ should be corrected by ‘to determine’.
- Introduction: First paragraph: One of the references in support of the 30% inappropriate antibiotic use is from the ambulatory care setting in the US, with data from 2010-2011. Perhaps a more suitable reference can be used here: more recent and from a closer geographical area?
- Introduction – Second paragraph: It seems relevant here to also discuss inappropriate use as a result of over the counter access/ non-prescription dispensing of antibiotics in LMICs/ or sub-Saharan Africa and/or Zimbabwe.
- Methods – Study design and participants. Could the authors provide a rationale for the chosen study design? Indeed, considering the study aim, a qualitative research approach involving in-depth interviews and/or focus groups would have been suitable and perhaps even be preferred. Did the SARS-CoV-2 pandemic play a role in the choice of the study design?
- Methods – Study design and participants. Could the authors provide more information on the selection of the 9 public health clinics? Were there only 9 public health clinics operational in the low income suburb of the Harare region at the time of the study? It is unclear how the 9 included PHCs relate to the 12 PHCs mentioned in the manuscript.
- Methods - Study participants: In the methods section the authors state that ‘The surveys were conducted during training sessions on AMR and antibiotic prescribing.’ Strikingly, the authors report in the result section ’Only one in three participants reported having received formal training on antibiotic prescribing in the previous year.’ This seems somewhat contradicting, how do the authors explain this? Are training sessions on AMR and antibiotic prescribing not considered formal training sessions? Is the distinction between formal training and informal training relevant and if so why? Did you also collect information on whether AMR and antibiotic stewardship /responsible antibiotic prescribing was part of the curriculum of the health care workers?
- Methods – Survey: Please add references for the other studies conducted in Zimbabwe mentioned.
- Methods—Survey: Four papers are listed as references for developing the survey. I notice that only one of them involved nurses as study participants [ref 7]. Could the authors please explain how they tailored the questions and content of the survey to their study population (i.e., nurses and midwives)? Were the survey questions piloted and or/ reviewed to assess whether the questions were suitable for the chosen study population?
- Methods - Data collection: As demographic data were also collected, ‘pseudonymised data’ seems a more appropriate term than ‘fully anonymised data’.
- Results - Prescriber knowledge: Consider clarifying why the clinical examples reported here are relevant to assess the knowledge of the prescribers. It might not be clear for everybody reading the manuscript that you are trying to highlight common inappropriate antibiotic use examples.
- Discussion - Paragraph 4: The authors mention here that patients could potentially report non existing symptoms with the goal to obtain antibiotics. This would suggest patients would have specific knowledge of symptom/treatment combinations. Also, the manuscript reports easy over the counter access of antibiotics for patients from pharmacies and informal markets. Altogether, exaggerating/faking symptoms seems a bit far stretched to me. I would suggest the authors to consider removing this point as it did not emerge from your own data.
- Discussion- Paragraph 7: ‘The survey focuses on nurses and midwives’ This sentence should be aligned with the aim as described in the last paragraph of the introduction (midwives are not listed there).
- Discussion – general: Focussing on midwives and nurses is actually an innovative and important aspect of the work. This should be emphasised more across the manuscript. For the discussion section, I suggest the authors add reflections on whether the findings of their work focussing on nurses and midwives actually identified new themes or topics as compared with literature focusing on medical doctors. Would the author recommend different or similar content for nurses and midwives as compared to medical doctors? Would they recommend other settings in which nurses and midwives are the main antibiotic prescriber to do similar research- and why? What type of data would be needed to ensure generalizability of the data for other prescribing nurses and midwives in Zimbabwe?
- Discussion –Strengths: I would suggest the authors emphasize the value of the study results. For example, results to be used for the design and shaping of future educational activities for local PHC staff (or more specifically nurses and midwives).

Is the work clearly and accurately presented and does it cite the current literature?

Partly

If applicable, is the statistical analysis and its interpretation appropriate?

Yes

Are all the source data underlying the results available to ensure full reproducibility?

Yes

Is the study design appropriate and is the work technically sound?

Partly

Are the conclusions drawn adequately supported by the results?

Partly

Are sufficient details of methods and analysis provided to allow replication by others?

Partly

Reviewer Expertise:

microbiology, antibiotic resistance, low resource settings, antibiotic use, social science

We confirm that we have read this submission and believe that we have an appropriate level of expertise to confirm that it is of an acceptable scientific standard, however we have significant reservations, as outlined above.

# Response

**Published:** 2022-04-26

**Authors:** Ioana Diana Olaru

**Affiliations:** London School of Hygiene & Tropical Medicine, UK

Thank you for reviewing our manuscript and for your suggestions. Please find below the responses to the issues raised.

**Reviewer summary:**

 The authors report on a cross-sectional survey exploring attitudes and practices of health care providers working in public primary health clinics in relation to microbiology tests, AMR and antibiotic use, in the Harare region in Zimbabwe. The choice of the research setting is especially of interest as nurses are the main antibiotics prescribers in public health clinics and literature on nurse prescribers is particularly scarce. Minor revisions would benefit the clarity of the manuscript and highlight the novelty of the work and thereby enhance its impact.

 Thank you. We agree with your comments and we have made the following changes in accordance to your suggestions:

**Minor Comments:**

**Comment #1**

 Abstract: Last sentence of the methods section: 'to determined’ should be corrected by ‘to determine’.

**Response to comment #1**

 Corrected.

**Comment #2**

 Introduction: First paragraph: One of the references in support of the 30% inappropriate antibiotic use is from the ambulatory care setting in the US, with data from 2010-2011. Perhaps a more suitable reference can be used here: more recent and from a closer geographical area?

**Response to comment #2**

 A reference was added in support of the statement - study by Fink et al. Lancet Infect Dis 2020 which includes six countries from sub-Saharan Africa. Also, the next paragraphs was re-worded to reflect potential differences between high- and low-resource settings. *“* *In high-income countries, more than 85% of antibiotics are prescribed in the community i.e. in outpatient settings* *^5^* *; this is likely similar in LMICs.”*

**Comment #3**

 Introduction – Second paragraph: It seems relevant here to also discuss inappropriate use as a result of over the counter access/ non-prescription dispensing of antibiotics in LMICs/ or sub-Saharan Africa and/or Zimbabwe.

**Response to comment #3**

 A comment was added according to the suggestion:

*“* *In many low-resource settings, non-prescription antibiotic use is a frequent phenomenon. ^24^ In Zimbabwe, antibiotic dispensing was historically highly regulated with only 8% of antibiotics issued without a prescription. ^25^ However, recent economic decline, increasing healthcare utilisation costs and the COVID-19 pandemic, has likely resulted in increased non-prescription antibiotic use.* *”*

**

**Comment #4**

 Methods – Study design and participants. Could the authors provide a rationale for the chosen study design? Indeed, considering the study aim, a qualitative research approach involving in-depth interviews and/or focus groups would have been suitable and perhaps even be preferred. Did the SARS-CoV-2 pandemic play a role in the choice of the study design?

**Response to comment #4**

 This study was embedded within the larger ARGUS study ( https://doi.org/10.12688/wellcomeopenres.15977.1) exploring the burden of antimicrobial resistance in community-acquired infections and antibiotic use in primary care using quantitative methods. Studies using in-depth interviews among healthcare workers and policy makers from Zimbabwe have already been conducted by our group (Dixon J et al. Soc Sci Med 2021). For the current study, we had originally planned to include a broader range of healthcare workers providing various levels of care (doctors and nurses from hospitals, doctors in training, medical students, pharmacists, etc.). However, the COVID-19 pandemic led to the cancellation of all in-person meetings and events and therefore we were not able to conduct the study among other groups of healthcare workers.

**Comment #5**

 Methods – Study design and participants. Could the authors provide more information on the selection of the 9 public health clinics? Were there only 9 public health clinics operational in the low income suburb of the Harare region at the time of the study? It is unclear how the 9 included PHCs relate to the 12 PHCs mentioned in the manuscript.

**Response to comment #5**

 The COVID-19 pandemic and the economic situation in Zimbabwe had a dramatic effect on healthcare provision. Many primary care clinics were closed and healthcare services were heavily disrupted. The study was conducted when some (but not all) of the clinics were resuming activities. The clinics were selected because they operated within low-income communities and they were open at the time when the study was conducted.

**Comment #6**

 Methods - Study participants: In the methods section the authors state that ‘The surveys were conducted during training sessions on AMR and antibiotic prescribing.’ Strikingly, the authors report in the result section ’Only one in three participants reported having received formal training on antibiotic prescribing in the previous year.’ This seems somewhat contradicting, how do the authors explain this? Are training sessions on AMR and antibiotic prescribing not considered formal training sessions? Is the distinction between formal training and informal training relevant and if so why? Did you also collect information on whether AMR and antibiotic stewardship /responsible antibiotic prescribing was part of the curriculum of the health care workers?

**Response to comment #6**

 Apologies for the apparent contradiction. The survey was done before the training and therefore the respondents commented on training before the current event. The current training was organised within dissemination/feedback activities of results from studies focusing on diagnosing infections and AMR conducted by our group. This was clarified in the methods in response to the comment of the other reviewer.

*“* *The questionnaires were self-administered using paper-based forms and was filled in prior to the session.”*

 Although we asked about sources of training for antimicrobial prescribing, we did not specifically ask about training on responsible prescribing. This is because prescriptions in primary care should follow the national treatment recommendations (EDLIZ). In practice, because of multiple reasons (diagnostic uncertainties, fears that patients may not be able to return if the condition becomes severe, feelings that some sort of care should be provided, etc.), the guidelines are not always followed (Dixon J, Soc Sci Med, 2021).

**Comment #7**

 Methods – Survey: Please add references for the other studies conducted in Zimbabwe mentioned.

**Response to comment #7**

 The other studies used different methodologies and did not have the specific aim of investigating knowledge, attitudes and practices of healthcare providers but rather provided a more comprehensive understanding of the landscape of antibiotic prescribing and antimicrobial resistance in Zimbabwe. A comment to that respect was added to the manuscript: “ *The studies did not assess the knowledge, attitudes and practices of healthcare workers but rather provided a more comprehensive understanding of the landscape of AMR and prescribing in Zimbabwe* *.” *These studies were also referenced in response to the comment of the other reviewer and some have been added to the manuscript text.

****

**Comment #8**

 Methods—Survey: Four papers are listed as references for developing the survey. I notice that only one of them involved nurses as study participants [ref 7]. Could the authors please explain how they tailored the questions and content of the survey to their study population (i.e., nurses and midwives)? Were the survey questions piloted and or/ reviewed to assess whether the questions were suitable for the chosen study population?

**Response to comment #8**

 The survey was originally planned to also include other categories of healthcare professionals providing different levels of care (doctors and nurses working in hospitals, medical students, pharmacists, etc.). For this, we originally designed two questionnaires according to the population being surveyed. These questionnaires had a number of common questions and also included questions specifically for inpatient settings (e.g. ordering blood cultures in hospitals). The COVID-19 pandemic led to severe disruption of healthcare provision in Zimbabwe. In-person meetings and teaching events were cancelled. As a result, the survey was conducted exclusively in primary health care clinics where nurses and midwives are the care providers. The questionnaires were reviewed by healthcare providers and researchers with expertise in evaluating antibiotic use prior to their use in the field. In Zimbabwe, primary care is provided almost universally by trained nurses which may not be the case in other settings (where doctors are the main prescribers).

**Comment #9**

 Methods - Data collection: As demographic data were also collected, ‘pseudonymised data’ seems a more appropriate term than ‘fully anonymised data’.

**Response to comment #9**

 Information on age and other variables that could potentially be used to identify individuals were collected in broad categories (e.g. age range rather than age or date of birth). No participant identifiers were given to the forms. Therefore data was anonymised.

**Comment #10**

 Results - Prescriber knowledge: Consider clarifying why the clinical examples reported here are relevant to assess the knowledge of the prescribers. It might not be clear for everybody reading the manuscript that you are trying to highlight common inappropriate antibiotic use examples.

**Response to comment #10**

 A clarification on the questions was added to the methods section:

*“* *The clinical questions were selected to reflect common scenarios that the nurses would encounter in their daily practice and might lead to inappropriate antibiotic use.”*

**

**Comment #11**

 Discussion - Paragraph 4: The authors mention here that patients could potentially report non existing symptoms with the goal to obtain antibiotics. This would suggest patients would have specific knowledge of symptom/treatment combinations. Also, the manuscript reports easy over the counter access of antibiotics for patients from pharmacies and informal markets. Altogether, exaggerating/faking symptoms seems a bit far stretched to me. I would suggest the authors to consider removing this point as it did not emerge from your own data.

**Response to comment #11**

 The section was removed according to the suggestion. It now reads: “ *However, there may be indirect pressures on the healthcare worker because they are aware that the patient may not be able to afford accessing the clinic again if symptoms become worse* *.”*

**Comment #12**

 Discussion- Paragraph 7: ‘The survey focuses on nurses and midwives’ This sentence should be aligned with the aim as described in the last paragraph of the introduction (midwives are not listed there).

**Response to comment #12**

 The aim of the study was amended to *“* *… explore attitudes and practices with regards to microbiology tests, AMR and antibiotic prescribing among healthcare providers (nurses and midwives) at public primary health clinics in Harare, Zimbabwe.”* In Zimbabwe, midwives are involved in providing antenatal and perinatal care including antibiotic prescriptions whenever deemed necessary. Some are dually trained and rotate into regular outpatient consultations.

**Comment #13**

 Discussion – general: Focussing on midwives and nurses is actually an innovative and important aspect of the work. This should be emphasised more across the manuscript.

**Response to comment #13**

 Thank you for this comment. We have further highlighted the novelty of surveying nurses and midwives on antibiotic prescribing throughout the manuscript:

*“The study used a new approach by focusing on nurses and midwives who are the main prescribers in outpatient settings in Zimbabwe.”* (Discussion, first paragraph).

*“* *Furthermore, the approach to survey nurses and midwives who are the main antibiotic prescribers in the public sector for outpatients is innovative.”* (Discussion, paragraph on study strengths).

**Comment #14**

 For the discussion section, I suggest the authors add reflections on whether the findings of their work focussing on nurses and midwives actually identified new themes or topics as compared with literature focusing on medical doctors.

**Response to comment #14**

 Comments were added on differences between findings from this study and those from studies conducted in other settings enrolling predominantly medical doctors.

*“Most healthcare providers indicated that the decision to prescribe antibiotics is mainly guided by the clinical presentation and the national guidelines and not directly by patient expectations. This is reassuring and contrary to findings from other settings where patients’ expectations played an important role in the decision to prescribe antibiotics.”. *(Discussion, comment already present in manuscript)

*“Furthermore in this study, the national guidelines were described as the main “influencer” in guiding antibiotic prescribing in routine practice. This is in contrast to a study from Gabon showing that prior experience and the opinion of the superior strongly influenced the decision whether or not antibiotics should be prescribed.” (Discussion)*

**Comment #15**

 Would the author recommend different or similar content for nurses and midwives as compared to medical doctors?

**Response to comment #15**

 We believe that the content of the survey has to be tailored to the study setting (country, type of facility) and the target population including their level of training and roles.

**Comment #16**

 Would they recommend other settings in which nurses and midwives are the main antibiotic prescriber to do similar research- and why?

**Response to comment #16**

 A sentence was added to the conclusion commenting on the need for further studies in settings where nurses and midwives are the main prescribers.

*“In many settings nurses and midwives are the main antibiotic prescribers. Hence understanding how to design training programmes for them is important. Surveys such as this one from other settings could potentially guide training and teaching programmes.”*

**Comment #17**

 What type of data would be needed to ensure generalizability of the data for other prescribing nurses and midwives in Zimbabwe?

**Response to comment #17**

 To make data more generalizable, one would need to conduct similar surveys among nurses and midwives working in the private sector and in rural communities. To our knowledge, in private clinics consultations are done by doctors and therefore nurses are not prescribers. A comment was added in the discussion *“These findings may not be generalizable to healthcare workers from private clinics or rural settings.”*

**Comment #18**

 Discussion –Strengths: I would suggest the authors emphasize the value of the study results. For example, results to be used for the design and shaping of future educational activities for local PHC staff (or more specifically nurses and midwives).

**Response to comment #18**

 Thank you for this suggestion. A comment was added in the discussion. *“* *The findings of this survey are of particular importance and can be used to inform the design of future educational activities for this group of healthcare professionals working in PHCs in Zimbabwe and elsewhere.”*

# Reviewer report: Reviewer response for version 1

**DOI:** 10.21956/wellcomeopenres.18368.r46817

**Published:** 2021-11-17

**Authors:** Clarence C Tam

**Affiliations:** 1 Saw Swee Hock School of Public Health, National University Singapore, Singapore, Singapore

The authors present the results of a survey of primary care prescribers in Harare on attitudes and practices related to antibiotic use. The findings are largely descriptive, as the modest size doesn’t allow for more in-depth or subgroup analyses. Nonetheless, the results are in line with findings from other settings and should provide useful information for guiding local practice, policy and further research in this important area. I have a few suggestions to improve the clarity of the work below.

**Major comments:**

1. It would be helpful if the authors could include a copy of the questionnaire as an appendix, and link this better with the information in figure 1. I appreciate the questionnaire is available in an online repository, but without ready access to this some of the information described in the paper is a bit difficult to interpret. In particular, it’s not easy to understand figure 1 without looking at how exactly the questions were asked and what the response options are, as the condensed labels in the figure don’t always give a good idea of what information is actually being presented.
2. Participants were recruited during training sessions on AMR and prescribing. Can the authors clarify when exactly in relation to this training participants completed the questionnaire? Was it before or after the training? It’s not clear at the moment whether participants’ responses reflect knowledge and experience prior to the training session, or information obtained or influenced by the training.
3. The survey questionnaire was informed by previous studies in Zimbabwe. Are these in the public domain? What specific information from these studies was used to develop the questionnaire?
4. What type of consent was obtained from participants? The methods section mentions that potential participants were provided with an information sheet with consent details, but it doesn’t mention whether consent was implied based on completion of the questionnaire, or obtained verbally, or whether written consent was sought. The latter wouldn’t necessarily constitute an anonymous survey, as stated in the discussion.
5. Can the authors clarify if the questionnaire was administered in English only, or if other languages were also used? Was any field testing of the questionnaire done to check that questions were understood by respondents as intended? I note from the questionnaire that some of the questions are not that easy to interpret. For example, one question asks:
*“In your practice, what is the importance of the following problems in affecting the use of microbiology results in your health facility?”*
  The first statement says *“* *Nurses are confident with empirical treatment and do not need the microbiology results for guidance”*, with response options ranging from “Very important” to “Very unimportant”. I don’t find this an intuitive question to answer, and it seems to me that there is scope for respondents not interpreting the question as intended.
6. Another concern I have with the questionnaire is that a number of the questions seem to be quite leading because of how they are asked or worded. It’s hard, for example, to disagree with a statement that says that ‘too many antibiotics are prescribed’. The same is true for ‘patient poor adherence', even though this might be contentious, particularly in a setting in which a large fraction of antibiotic use might be inappropriate or unnecessary in the first place. I suspect the response distributions might have been somewhat different if more neutral wording had been used, or if the questions had been asked in a different way, e.g. by asking participants to rank the top X causes of AMR. Another common practice is to insert statements that are not relevant to the question, as a check that responses aren’t unduly influenced by potential bias in the wording. From the current question, I don’t think it’s easy to distinguish whether the question is capturing the extent to which respondents agree with a statement or the extent to which they think a factor is an important driver of resistance. I don’t think there’s a fix for this here, but to me, it’s a limitation.
7. Another potential limitation might be the generalisability of findings from this study to primary care centres more broadly, given the profile of centres included in this research. Perhaps the authors could comment on this in the discussion.

**Minor comments:**

1. In the introduction, paragraph 2, I would clarify this sentence: “The vast majority of antibiotics are prescribed by practitioners in outpatient settings”. Do the authors mean that the majority of antibiotic prescriptions are issued in outpatient settings? Many, perhaps most, antibiotics in some settings are not prescribed but obtained over the counter or through informal sources.
2. I think it’s important to temper or contextualise this sentence in the concluding paragraph: “these findings support the need for increased availability of laboratory testing and for educational interventions and practice guidelines to limit overuse of antibiotics.” It’s hard to argue against the need for any of these things, but the reality is that even if the infrastructure and technology to facilitate more widespread testing as a diagnostic and prescribing aid is available, with a few exceptions such as malaria and dengue RDTs, currently the ability to do these within a clinically relevant timeframe is limited even in high-resource settings, and the ability and willingness of patients to undergo such testing is likely to be low, particularly if the financial cost is higher than the cost of antibiotics.

Is the work clearly and accurately presented and does it cite the current literature?

Yes

If applicable, is the statistical analysis and its interpretation appropriate?

Yes

Are all the source data underlying the results available to ensure full reproducibility?

Yes

Is the study design appropriate and is the work technically sound?

Partly

Are the conclusions drawn adequately supported by the results?

Partly

Are sufficient details of methods and analysis provided to allow replication by others?

Partly

Reviewer Expertise:

infectious disease epidemiology, antibiotic resistance, socio-behavioural research

I confirm that I have read this submission and believe that I have an appropriate level of expertise to confirm that it is of an acceptable scientific standard, however I have significant reservations, as outlined above.

# Response

**Published:** 2022-04-26

**Authors:** Ioana Diana Olaru

**Affiliations:** London School of Hygiene & Tropical Medicine, UK

We are very thankful for your comments and suggestions. Please find here the point-by-point responses to your comments.

**Comment #1**

 It would be helpful if the authors could include a copy of the questionnaire as an appendix, and link this better with the information in figure 1. I appreciate the questionnaire is available in an online repository, but without ready access to this some of the information described in the paper is a bit difficult to interpret. In particular, it’s not easy to understand figure 1 without looking at how exactly the questions were asked and what the response options are, as the condensed labels in the figure don’t always give a good idea of what information is actually being presented.

**Response to comment #1**

 Thank you for your suggestion. Unfortunately, it is not possible to have supplementary materials for articles submitted to Wellcome Open Research and additional materials are either included in the manuscript or deposited in online repositories. This is the case for this manuscript where the questionnaire was made available within Dryad *doi:10.5061/dryad.66t1g1k1s.*

**Comment #2**

 Participants were recruited during training sessions on AMR and prescribing. Can the authors clarify when exactly in relation to this training participants completed the questionnaire? Was it before or after the training? It’s not clear at the moment whether participants’ responses reflect knowledge and experience prior to the training session, or information obtained or influenced by the training.

**Response to comment #2**

 Questionnaires were distributed and filled prior to the sessions. A clarification was added to the manuscript text *“* *The questionnaires were self-administered using paper-based forms and was filled in prior to the session.”*

**Comment #3**

 The survey questionnaire was informed by previous studies in Zimbabwe. Are these in the public domain? What specific information from these studies was used to develop the questionnaire?

**Response to comment #3**

 The questionnaires were informed by our work within several other studies that were conducted in public healthcare facilities in Harare, Zimbabwe. While these studies do not use the same approach, they provided ample opportunity to identify day-to-day challenges that healthcare providers face in accessing diagnostics and prescribing treatment as well as highlight the problem of antimicrobial resistance. This knowledge was used to design the questionnaire for this study. The study protocols and some of the findings from these studies are already published (see below). In addition, the questions were informed by other studies from sub-Saharan Africa as described in the manuscript text.

*Hopkins H, et al. Febrile Illness Evaluation in a Broad Range of Endemicities (FIEBRE): protocol for a multisite prospective observational study of the causes of fever in Africa and Asia. BMJ Open. 2020 Jul 21;10(7):e035632. doi: 10.1136/bmjopen-2019-035632.*

*Olaru ID, et al. Antimicrobial Resistance in Gram-negative bacteria from Urinary Specimens: a study of prevalence, risk factors and molecular mechanisms of resistance (ARGUS) in Zimbabwe - a study protocol. Wellcome Open Res, 2020 Jun 12;5:140.*

*Chimhini G, et al. Evaluation of a Novel Culture System for Rapid Pathogen Identification and Detection of Cephalosporin Resistance in Neonatal Gram-negative Sepsis at a Tertiary Referral Unit in Harare, Zimbabwe. Pediatr Infect Dis J. 2021 Sep 1;40(9):785-791.*

*Olaru ID, et al. Assessment of antimicrobial use and prescribing practices among pediatric inpatients in Zimbabwe. J Chemother. 2020 Mar 2:1-4. doi: 10.1080/1120009X.2020.1734719.*

*Dixon J, et al. Antibiotics, rational drug use and the architecture of global health in Zimbabwe. Soc Sci Med. 2021 Mar;272:113594. doi: 10.1016/j.socscimed.2020.113594. Epub 2020 Dec 11.*

**Comment #4**

 What type of consent was obtained from participants? The methods section mentions that potential participants were provided with an information sheet with consent details, but it doesn’t mention whether consent was implied based on completion of the questionnaire, or obtained verbally, or whether written consent was sought. The latter wouldn’t necessarily constitute an anonymous survey, as stated in the discussion.

**Response to comment #4**

 The study was initially introduced by the lead researcher and healthcare workers were asked if they were interested to participate. Following that, the questionnaire was distributed which contained an information section asking the participants to fill in and return the survey if they consented to participate. No names or any other information that could be used to identify individuals were recorded on the answer sheet and therefore it was anonymous.

**Comment #5**

 Can the authors clarify if the questionnaire was administered in English only, or if other languages were also used? Was any field testing of the questionnaire done to check that questions were understood by respondents as intended? I note from the questionnaire that some of the questions are not that easy to interpret. For example, one question asks:

*“In your practice, what is the importance of the following problems in affecting the use of microbiology results in your health facility?”*

 The first statement says *“Nurses are confident with empirical treatment and do not need the microbiology results for guidance”*, with response options ranging from “Very important” to “Very unimportant”. I don’t find this an intuitive question to answer, and it seems to me that there is scope for respondents not interpreting the question as intended.

**Response to comment #5**

 The questionnaires were administered in English which is commonly used for professional communication. The questions were based on questionnaires used in other similar studies from sub-Saharan Africa. Prior to the study, the questionnaires were reviewed by other researchers and healthcare providers with expertise in antibiotic prescribing and resistance to determine if the questions are clear. We acknowledge that some questions may have been more difficult to understand and a comment to that respect was added to the limitations section.

*“Participants may have misunderstood some of the questions however the questions were informed by questionnaires used in other studies from sub-Saharan Africa and responses were generally consistent.”*

**

**Comment #6**

 Another concern I have with the questionnaire is that a number of the questions seem to be quite leading because of how they are asked or worded. It’s hard, for example, to disagree with a statement that says that ‘too many antibiotics are prescribed’. The same is true for ‘patient poor adherence', even though this might be contentious, particularly in a setting in which a large fraction of antibiotic use might be inappropriate or unnecessary in the first place. I suspect the response distributions might have been somewhat different if more neutral wording had been used, or if the questions had been asked in a different way, e.g. by asking participants to rank the top X causes of AMR. Another common practice is to insert statements that are not relevant to the question, as a check that responses aren’t unduly influenced by potential bias in the wording. From the current question, I don’t think it’s easy to distinguish whether the question is capturing the extent to which respondents agree with a statement or the extent to which they think a factor is an important driver of resistance. I don’t think there’s a fix for this here, but to me, it’s a limitation.

**Response to comment #6**

 Thank you for the comment. We agree that these factors may have influenced responses although it is in line with general perceptions (outside the findings of this study) that antibiotics are overprescribed. According to your suggestion, we added a statement in the limitations section of the discussion: *“* *Also, responses to some questions may be difficult to interpret because the respondent may have answered in the same way if they agreed with a statement or thought the was important.*

**Comment #7**

 Another potential limitation might be the generalisability of findings from this study to primary care centres more broadly, given the profile of centres included in this research. Perhaps the authors could comment on this in the discussion.

**Response to comment #7**

 Generalisability is further discussed in the limitations section. Because data were collected from nine primary care clinics across Harare, which represent most of the facilities serving low-income communities, we feel that data are generalisable to healthcare workers from this setting. We are unclear what the reviewer means with “profile of the centres”. The primary care clinics where health care workers were samples from are public health care facilities. We fully agree that data may not be generalisable to healthcare workers working in rural communities and in the private sector and a comment was added to this respect in the discussion.

*“These findings may not be generalizable to healthcare workers working in private clinics or rural settings.”*

**Minor comments:**

**Comment #8**

 In the introduction, paragraph 2, I would clarify this sentence: “The vast majority of antibiotics are prescribed by practitioners in outpatient settings”. Do the authors mean that the majority of antibiotic prescriptions are issued in outpatient settings? Many, perhaps most, antibiotics in some settings are not prescribed but obtained over the counter or through informal sources.

**Response to comment #8**

 The sentence was clarified according to your suggestion *“* *In high-income countries, more than 85% of antibiotics are prescribed in the community i.e. in outpatient settings* *^5^* *; this is likely similar in LMICs.”*

 Also a sentence on non-prescription antibiotic use was included: “ *In many low-resource settings, non-prescription antibiotic use is a frequent phenomenon. ^24^ In Zimbabwe, antibiotic dispensing was historically highly regulated with only 8% of antibiotics issued without a prescription. ^25^ However, recent economic decline, increasing healthcare utilisation costs and the COVID-19 pandemic, has likely resulted in increased non-prescription antibiotic use* *.”*

**Comment #9**

 I think it’s important to temper or contextualise this sentence in the concluding paragraph: “these findings support the need for increased availability of laboratory testing and for educational interventions and practice guidelines to limit overuse of antibiotics.” It’s hard to argue against the need for any of these things, but the reality is that even if the infrastructure and technology to facilitate more widespread testing as a diagnostic and prescribing aid is available, with a few exceptions such as malaria and dengue RDTs, currently the ability to do these within a clinically relevant timeframe is limited even in high-resource settings, and the ability and willingness of patients to undergo such testing is likely to be low, particularly if the financial cost is higher than the cost of antibiotics.

**Response to comment #9**

 Thank you for your comment.

 To better reflect this, the sentence in the conclusion was amended according to the recommendations: *“While increased availability of diagnostics would be ideal, this is unlikely to materialise due to financial and infrastructural constraints. Also in reality turnaround times of microbiological diagnostics is usually too long and hence has limited impact on patient management, specifically in outpatient settings. However, establishing sentinel sites to determine causative organisms in certain settings and generate data on AMR might be a possible solution.”*
